# Phenotypic bacterial epidemiology and antimicrobial resistance profiles in neonatal sepsis at Jimma medical center, Ethiopia: Insights from prospective study

**DOI:** 10.1371/journal.pone.0310376

**Published:** 2024-09-16

**Authors:** Daniel Geleta, Gemeda Abebe, Tsion Tilahun, Didimos Gezahegn, Netsanet Workneh, Getenet Beyene

**Affiliations:** 1 Department of Medical Laboratory Sciences, Jimma University, Jimma, Oromia, Ethiopia; 2 Mycobacteriology Research Center, Jimma University, Jimma, Oromia, Ethiopia; 3 Department of Pediatrics and Child Health, Jimma University, Jimma, Oromia, Ethiopia; 4 Microbiology Unit, Jimma Medical Center, Jimma, Oromia, Ethiopia; 5 Department of Health Behavior and Society, Jimma University, Jimma, Oromia, Ethiopia; Debre Markos University, ETHIOPIA

## Abstract

**Background:**

Epidemiological profiles and the rundown crisis of antimicrobial resistance from bacterial isolates in neonatal sepsis compel regular surveillance to enhance data-driven decision-making. Accordingly, this study aimed to assess the phenotypic epidemiology and antimicrobial resistance profiles of bacteria isolated from clinically suspected neonatal sepsis in Ethiopia.

**Methods:**

A total of 342 neonates suspected of clinical sepsis were randomly included in a prospective observational study conducted at the neonatal intensive care unit (NICU) of Jimma medical center (JMC) from May 2022 to July 2023. Blood samples were collected from each neonate and subjected to a culture test for identification of bacterial isolates and their antibiotic resistance profiles following the standardized guidelines. The laboratory results, along with relevant clinical data, were recorded using WHONET and analyzed using STATA software.

**Results:**

Out of the 342 blood samples that were analyzed, 138 samples (40.4%, 95% CI: 35.1–45.6, P<0.01) exhibited proven bacterial infection. The infection rates were notably higher in males with 85/138 (61.6%, 95% CI: 53.4–69.8, P<0.01) and neonates aged 0–3 days with 81/138 (58.7%, 95% CI: 50.5–66.9, P<0.01). The majority of the infections were attributed to Gram-negative bacteria, accounting for 101/138(73.2%, 95% CI: 65.6–80.7) cases, with 69/101(68.3%, 95% CI: 63.8–72.8) cases involving ESBL-producing strains, while Gram-positive bacteria were responsible for 26.8% (95% CI: 19.3–34.4) of the infections. The predominant isolates included *Klebsiella pneumoniae* (37.7%, 95% CI: 29.6–45.8), *Coagulase-negative Staphylococci* (CoNs) (20.3%, 95% CI: 13.6–27.0), and Acinetobacter species (11.6%, 95% CI: 6.0–17.1). Of the total cases, 43/72 (59.7%, 95% CI: 48.4–71.1, P<0.01) resulted in mortality, with 28/72 (38.9%, 95% CI: 27.70–50.1, P<0.03) deaths linked to Extended-Spectrum Beta-Lactamase (ESBL)-producing strains. *Klebsiella pneumoniae* displayed high resistance rates to trimethoprim-sulfamethoxazole (100%), ceftriaxone (100%), cefotaxime (98.1%), ceftazidime (90.4%), and gentamicin (84.6%). Acinetobacter species showed resistance to ampicillin (100%), cefotaxime (100%), trimethoprim-sulfamethoxazole (75%), ceftazidime (68.8%), chloramphenicol (68.8%), and ceftriaxone (68.8%). Likewise, *CoNs* displayed resistance to ampicillin (100%), penicillin (100%), cefotaxime (86.0%), gentamicin (57.2%), and oxacillin (32.2%). Multidrug resistance was observed in 88.4% (95% CI: 81.8–93.0) of isolates, with ESBL-producers significantly contributing (49.3%, 95% CI: 45.1–53.5). Furthermore, 23.0% (95% CI: 15.8–31.6) exhibited a prevalent resistance pattern to seven distinct antibiotic classes.

**Conclusion:**

The prevalence and mortality rates of neonatal sepsis were significantly high at JMC, with a notable surge in antibiotic and multidrug resistance among bacterial strains isolated from infected neonates, specifically ESBL-producers. These resistant strains have a significant impact on infection rates and resistance profiles, highlighting the requisite for enhanced diagnostic and antimicrobial stewardship, stringent infection control, and further molecular characterization of isolates to enhance neonatal survival.

## Introduction

Neonatal sepsis presents a major global public health issue, affecting millions of neonates worldwide each year and worsened by antimicrobial resistance [1]. It consists of two distinct categories: early-onset (days of life 0–3) and late-onset (days of life 4 or later), determined by timing, transmission mode, and causative organisms [2]. Bacteria are the primary culprits in categories, contributing to the fragile epidemiology of the infection, and exacerbating the rundown global crisis of antimicrobial resistance [3].

In the battle against this crisis, blood culture testing remains the gold standard for identifying bacterial causes and guiding treatment options. However, the ongoing preference for less accurate clinical diagnostic methods over blood culture testing complicates the epidemiology of neonatal sepsis [4]. However, the persistent preference for less accurate clinical diagnostic approaches over blood culture testing complicates the epidemiology of neonatal sepsis [5]. Consequently, the global prevalence of neonatal sepsis exhibits significant variation, with rates ranging from 25% to 60% across different neonatal populations worldwide [6, 7]. This variability is influenced by the diagnostic methods employed, the types of bacteria implicated, and the specific geographic locations where these cases are observed [8]. For instance, in regions like Saudi Arabia and Bangladesh, neonatal sepsis commonly involves both Gram-positive and Gram-negative bacterial strains, including Enterobacter, *Escherichia coli (E*. *coli)*, *Klebsiella pneumoniae (K*. *pneumoniae)*, *Acinetobacter baumannii*, and *Group B Streptococcus* (*GBS*) [9, 10]. Similarly, in countries like Pakistan, Egypt, and India, prevalent pathogens contributing to neonatal sepsis include *Salmonella Typhi*, *Coagulase-negative staphylococci (CoNs)*, and *Staphylococcus aureus (S*. *aureus)*, respectively [11–13]. In Jimma, Ethiopia, the primary pathogens identified in neonatal sepsis cases are Klebsiella species (36.6%), *CoNS*, 19.7%), and *S*. *aureus* (18.3%) [14].

Notably, the bacteria commonly encountered in healthcare settings often exhibit resistance to first-line antibiotics used in the treatment of sepsis, such as ampicillin, gentamicin, and cefoxitin. In less developed areas, a significant percentage of these bacteria demonstrate resistance, with rates ranging from 50% to 88% [8]. Notably, strains of *E*. *coli*, *(S*. *aureus)*, and *K*. *pneumoniae* exhibit significant resistance to antibiotics such as amoxicillin, cephalosporins, aminoglycosides, and quinolones across several countries [10]. Within Ethiopian hospitals, including the current study setting, Gram-positive bacteria often show high resistance rates to penicillin (98.9%) and ceftriaxone (91.3%), while Gram-negative bacteria display resistance to ampicillin (100%), gentamicin (83.2%), and ceftriaxone (83.2%) [15].

Moreover, the rise in antimicrobial resistance, driven by various bacterial mechanisms, continues to pose a catastrophic public health threat [16]. Among these threats, the emergence and dissemination of Extended-Spectrum Beta-Lactamase (ESBL) strains and the increase in multidrug-resistant (MDR) strains created an ominous future [17, 18]. These strains demonstrate a delicate epidemiology that varies across regions, settings, and even among different strains, with a rising global prevalence of ESBL-producing and MDR strains [17]. For instance, the prevalence of ESBL-producing isolates has been reported at 42.7% in China [19], 98% in Zambia [20] and 54% in Ethiopia [21]. Similarly, MDR has been reported at 64.7% in China [19], 70% in Tanzania [22] and 84% in a previous study conducted in Ethiopia [23] indicating spiraling global spread [1].

Consequently, the global partners are advocating for collective action to combating sepsis by focusing on various key areas. These include detecting the causative pathogens, monitoring trends in antimicrobial resistance, developing effective infection control measures, improving diagnostic capabilities, and identifying emerging threats worldwide. This concerted effort aims to alleviate the global threat posed by sepsis and safeguard future generations through enhanced detection and effective management [24]. Despite these efforts, statistical data consistently highlights the significant role of bacterial pathogens in the onset of neonatal sepsis, compounded by the challenge of antimicrobial resistance. Concerningly, bacterial isolation and antimicrobial resistance profiling are often limited or inconsistent in many countries, including Ethiopia. Therefore, this study seeks to delve into the phenotypic epidemiology and antimicrobial resistance profiles of bacterial isolates in neonatal sepsis.

## Materials and methods

### Study design, period and setting

A descriptive cohort study was executed at the Jimma Medical Center (JMC), a tertiary and referral teaching medical center in Ethiopia, from May 1, 2022 to July 31, 2023. JMC serves approximately 15 million outpatients and 16,000 inpatients annually across its various departments, which operate 24 hours a day. The pediatric department, which manages a pediatric and neonatal intensive care unit (PNICU), admits an average of 75 neonates per month for various complaints and provides routine neonatal care services. Neonates in the PNICU were diagnosed with sepsis by a pediatrician using national guidelines and suggested laboratory parameters [25]. Samples for recommended laboratory tests were collected by trained nurses and transferred to the main laboratory department of the center, which is situated around 200 meters away from the PNICU. The transferred samples were checked for quality, given a specific code, and processed, with particular blood culture and antimicrobial sensitivity tests (AST) performed in a well-equipped and internationally accredited microbiology sub-unit. The individual patient records of the center, including demographics, diagnosis, treatment, and treatment outcomes, were summarized monthly and reported to the Health Information Management System office, which utilizes the District Health Information System software to store the data.

### Study population, sample size estimation and sampling technique

The sampling of neonates admitted to the JMC NICU before their 28^th^ birthday was predated, and eligible subjects of either sex were included using a systematic random sampling approach. The study used STATCAL and the single proportion formula to determine the required sample size of 342 neonates, considering a 36% prevalence of neonatal bacteremia, a 95% CI, and a 5% margin of error [26, 27]. The research team employed a random selection process to identify neonates with sepsis who exhibited at least one of the clinical signs: fever (>38°C) or hypothermia (<36°C), rapid breathing (>60 breaths/minute), severe chest indrawing, poor feeding, seizure, lethargy, or unconsciousness, and two hematologic criteria: total leukocyte count <5000 or >12,000 cells/m3, absolute neutrophil count <1500 cells/mm3 or >7500 cells/mm3, ESR >15/h, and platelet count <150,000 or >440,000 cells/mm3)] [28]. The study involved close (every hour) monitoring of selected participants within the first 6 hours of hospital admission, followed by daily monitoring throughout their inpatient stay or for up to 28 days. During the monitoring period, the study team documented the index date for each participant based on assessments and assertions made by the ward pediatrician (death, or improvement).

### Inclusion criteria

The study included all neonates with suspected clinical sepsis eligible for antibiotic treatment, with documented blood culture results, and treatment outcomes.

### Exclusion criteria

The study excluded neonates with incomplete records, severe congenital anomalies, pre-enrollment antibiotic treatment, antibiotic allergies, contaminated blood samples, and non-sepsis-related deaths.

### Data source, data collection tool & procedure

The study utilized an Android-based Kobo-Collect application and a validated questionnaire to collect data from neonatal guardians, primarily mothers. The data collection process involved face-to-face interviews conducted by four BSc nurses after obtaining formal written consent from the parent or guardian of neonates. The collected data covered socio-demographics, obstetrics and delivery details, and neonatal factors. Finally, the collected data were merged with the specific laboratory data of individual neonatal participants that was uploaded into the WHONET software during the laboratory work.

### Blood sample collection and pathogen profiling

A trained and experienced phlebotomist expertly extracted 1–3 ml of blood from various peripheral veins, commonly in the arm, employing strict aseptic techniques. The obtained blood samples were then introduced into Brain Heart Infusion Broth bottles using a double-needle method. Each sample was meticulously labeled and promptly transported to the microbiology laboratory for immediate processing. In the laboratory, the samples were cultured on Mac-Conkey Agar and blood agar plates (supply of Liofilchem®, Italy). The culture plates were incubated in an aerobic environment at 37°C and monitored daily for seven days, with observations made within a time frame of 18–24 hours. For colonies observed on the plates, further analysis was performed for identification of the specific bacterial types. To identify Gram-positive isolates, a range of methods outlined in the CLSI 2023 guidelines were employed, including Gram staining, observation of colony characteristics, and assessment of biochemical properties such as catalase, DNAse agar, Mannitol Salt Agar, and hemolysis on blood agar plates. On the other hand, Gram-negative bacilli were further identified through various biochemical tests, including triple sugar iron (TSI), lysine iron agar (LIA), motility in indole, ornithine (MIO), citrate, urease, and oxidase enzyme production. Blood culture bottles that showed bacterial growth and were consistent with the symptoms of neonatal infection were considered positive. However, if a neonate’s clinical symptoms were not consistent with a positive blood culture, particularly for coagulase-negative Staphylococcus or Staphylococcus aureus, the attending pediatrician concluded that the positive result was due to contamination [29].

### Antimicrobial susceptibility testing

The susceptibility of the identified bacteria to antimicrobial agents was assessed in accordance with the Clinical and Laboratory Standards Institute (CLSI) 2023 guideline [29]. The Kirby-Bauer disk diffusion method, which involved placing antimicrobial disks on specific types of agar, mostly Muller-Hinton agar supplemented with 5% defibrinated sterile sheep blood, was used. The selection of antimicrobial agents was based on the prevalence of bacteria, commonly used antibiotics in the study setting, and laboratory protocols for isolates from sepsis patients. The selected antimicrobial agents consisted of eight different classes: penicillin (PEN), beta-lactamase inhibitors (BLI), sulfonamide (SUL), phenols (PHE), fluoroquinolones (QNL), aminoglycosides (AMN), cephalosporins (CEP), and carbapenems (CAR). Within each class, specific agents were used, including Ampicillin (AMP: 105 μg), Benzyl Penicillin (PEN: 10 μg), Oxacillin (OXA: 1 μg), Sulfamethoxazole-trimethoprim (SXT: 3.75/1.25 μg), Amoxicillin-Clavulanate (AMC: 20 μg), Tazobactam (TZP: 10 μg), Chloramphenicol (CHL: 30 μg), Ciprofloxacin (CIP: 5 μg), Gentamicin (GEN: 10 μg), Vancomycin (VAN: 30 μg), Cefotaxime (CTX:30 μg), Ceftazidime (CAZ: 30 μg), Ceftriaxone (CRO: 30 μg), Cefoxitin (FOX: 30 μg), and Meropenem (MEM: 20 μg). The bacterial isolates were cultured on plates and then incubated either in an aerobic or microaerophilic environment for a specific duration, typically 24–48 hours, depending on the requirements of each organism. After incubation, the zones of inhibition, which indicate areas where bacterial growth is inhibited by the antimicrobial discs, were measured using a caliper in millimeters. The measurements were recorded in a WHONET template, which used specified breakpoints to categorize isolates as susceptible, intermediate, or resistant, per CLSI 2023 guidelines [29].

### Phenotypic screening and confirmation of ESBLs

In this study, Gram-negative bacterial isolates were investigated, focusing on those with small inhibition zones when exposed to ceftazidime 30 μg (≤22mm), Ceftriaxone 30μg(≤25mm) or cefotaxime (30 μg) (≤27mm). The isolates were initially identified using a combination disc screening test and confirmed with a double disc diffusion test. The tests involved placing discs containing ceftazidime 30 μg or cefotaxime 30 μg alone and ceftazidime or cefotaxime 30 μg with clavulanic acid on a bacterial culture. An increased zone of inhibition (≥5mm) with the combined disc when compared to a single ceftazidime or cefotaxime disc indicated the phenotypic evidence of extended-spectrum beta-lactamase (ESBL) production. Positive and negative controls, including ESBL-producing *Klebsiella pneumonia* ATCC 700603 and non-ESBL-producing *E*. *coli* ATCC 25922 control strains, were used during the laboratory analysis to ensure the reliability of the results [29].

### Data quality management and control

Data quality was assured through the translation of the data collection tool into local languages, specifically Afaan-Oromo and Amharic, and then back-translation to the original version. Using the translated tool, a trial run of the tool was performed on 5% of a similar population outside the study facility before the actual data collection. Subsequently, the validated and configured data tools were integrated into Kobocollect, and all data collectors underwent comprehensive two-day training. The questionnaire was loaded onto their Android devices, making data collectors well-prepared and acquainted with the tools. During the data collection process, the researcher regularly reviewed the collected and uploaded data to ensure accuracy, completeness, clarity, and consistency and to uphold the quality and dependability of the data.

Likewise, the laboratory kits, media, antibiotic discs, and other consumables were purchased from a reputable and standardized supplier. Before using the in-house-prepared culture media plates for real samples, each batch underwent sterilization checks. The laboratory analysis strictly adhered to standard operating procedures to maintain consistency and accuracy. Control strains, specifically *S*. *aureus* (ATCC-25923) for Gram-positive bacteria and *P*. *aeruginosa* (ATCC-27853) and *E*. *coli* 35218 for Gram-negative bacteria, were employed for the purpose of isolate detection and antimicrobial susceptibility testing. Before the actual data analysis, data profiling was conducted using frequency distributions and cross-tabulations.

### Ethical issues

The research study obtained approval from the Jimma University Institute of Health Science Review Board on February 9, 2022, under reference number JUIRB32/2022. Following this, it was granted permission by the Jimma Medical Center Ethical Committee on February 22, 2022, with reference number THRPGn/344/2022. In adherence to the Helsinki Declaration, the researchers obtained written informed consent from the primary guardian of each neonate prior to data collection, ensuring compliance with the established protocol.

### Data analysis

The data management process effectively utilized two powerful tools, STATA version 16.0 and WHONET 2022 software. WHONET, specialized software for analyzing antimicrobial resistance patterns in microbiology data, was employed to store and interpret antimicrobial resistance breakpoints and perform interim analysis of resistance profiles. Concurrently, STATA software was used to provide comprehensive descriptions of socio-demographic patterns, bacterial isolate epidemiology, and antimicrobial resistance profiles. Statistical techniques were applied to express categorical variables through absolute frequencies (N), percentages (%), confidence levels (CI), and visualizations. The results of the analyses were effectively communicated using well-designed tables, figures, and informative narrative texts.

## Results

### Socio-demographic and clinical profile of participants

The study recruited a cohort of 342 neonates from rural (51.2%) and urban (48.8%) settings. Among these neonates, the majority (75.1%) were born to mothers aged 20–34 years, with males making up 60.2% of the group. A significant number (57.6%) of neonates were admitted within the initial 3 days of life. Roughly 68.1% of neonates were born at or after 36 weeks of gestation, hinting at a preterm birth rate among the rest. The vast majority (89.2%) of births occurred in healthcare facilities, with a substantial portion (74.6%) being non-cesarean deliveries compared to 25.1% delivered via cesarean section. Additionally, 72.5% of neonates had a birth weight of ≥ 2500 grams, while 27.5% were classified as low birth weight. Early-onset sepsis was identified in 65.5% of neonates, while late-onset sepsis affected 34.5% of cases. Concerning the source of infection, 53.5% were community-acquired, while 46.5% were hospital-acquired. Upon admission, all neonates received empirical antibiotic therapy, primarily consisting of ampicillin and gentamicin (59.6%). Eventually, 78.9% of neonates showed improvement and were discharged, while 21.1% risked sepsis attributed death (Table 1).

**Table 1:** Socio-demographic & clinical characteristics of the participant neonates (N = 342) at JMC, Ethiopia.

Variable	Categories	Frequency	Percentage
Residence	rural	175	51.2
	Urban	167	48.8
Maternal age	≤19 years	54	16.1
	20–34 years	257	75.1
	≥35 years	31	9.1
Sex of the neonate	Male	206	60.2
	Female	136	39.8
Age on admission (days)	0–3	200	58.5
	≥4	142	41.5
Birth order in the family	First	133	38.9
	2–3	128	37.4
	≥4	81	23.7
Gestational age	≥37 weeks	233	68.1
	Bellow 37 weeks	109	31.9
Place of delivery	Health facility	298	89.2
	Home	37	10.8
Mode of delivery	Non-cesarean section	256	74.9
	Cesarean section	86	25.1
Intrapartum antibiotic prophylaxis	Yes	146	42.7
	No	196	57.3
Weight at birth	Normal (≥2500gm)	248	72.5
	Low(<2500gm)	94	27.5
Type of sepsis	Early onset	221	65.5
	Late onset	121	34.5
Possible place of infection	Hospital	159	46.5
	Community	183	53.5
Empirical antibiotic regimen	Ampicillin + Gentamicin	204	59.6
	Ceftriaxone + Gentamicin	81	23.7
	Ceftazidime + Vancomycin	52	15.2
	Others *	5	1.4
Neonatal outcome at discharge or end of follow up	Improved	270	78.9
	Died	72	21.1

Others

* Ampicillin + Vancomycin (3), Ceftazidime & Metronidazole (2), Improved: a positive change in the neonates’ health status, signaling progress and recovery in response to treatment (stable vital signs, improved biomarkers and feeding).

### Epidemiology of bacterial isolates in neonatal sepsis

**Fig 1:**
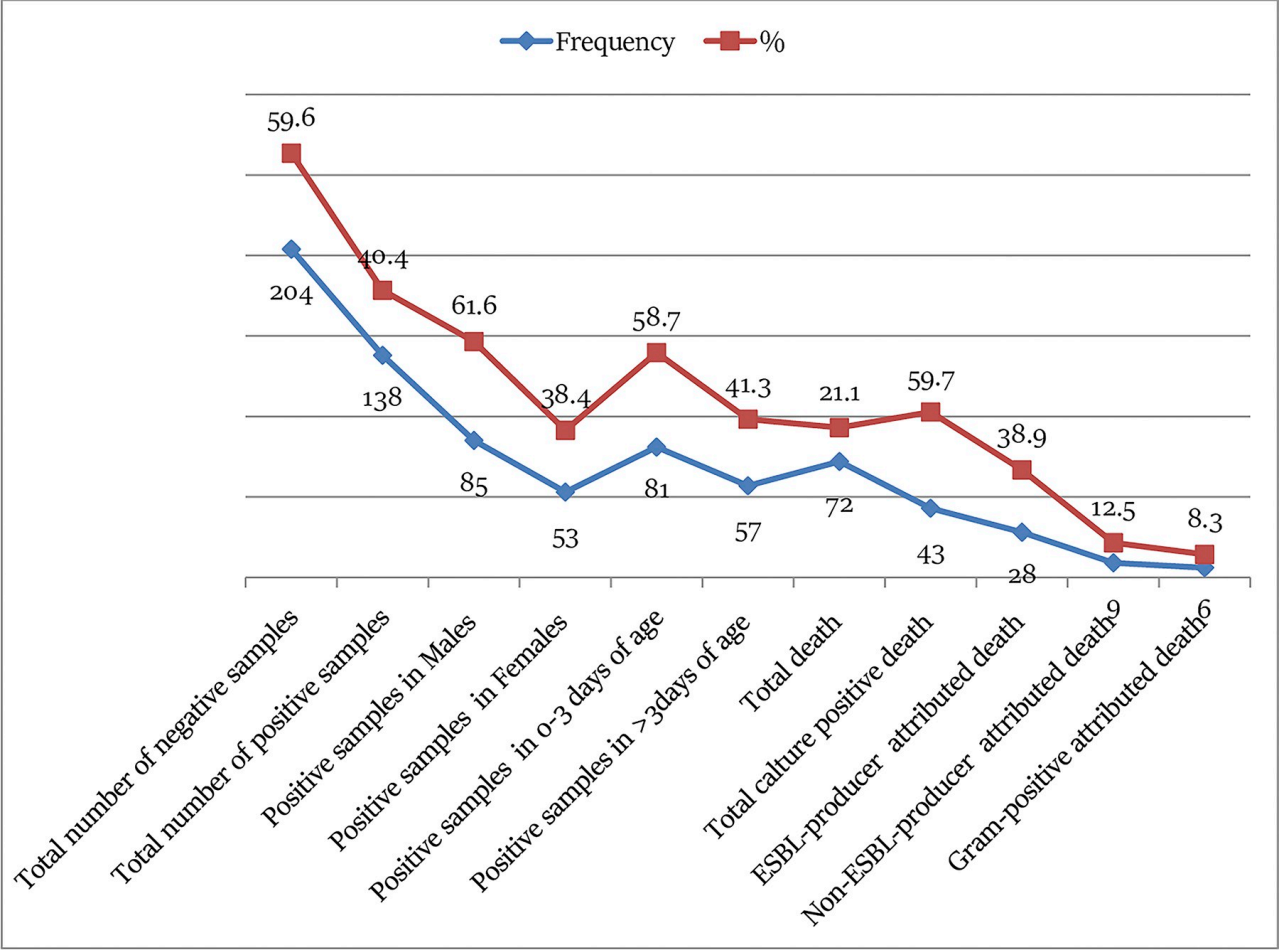
Distribution of positive blood culture within risk factors and treatment outcomes in neonatal sepsis at JMC in Ethiopia.

Out of the 342 blood samples that were analyzed, 138 samples (40.4%, 95% CI: 35.1–45.6, P<0.01) exhibited proven bacterial infection. The infection rates were notably higher in males with 85 out of 138 samples (61.6%, 95% CI: 53.4–69.8, P<0.01) and in neonates aged 0–3 days with 81 out of 138 samples (58.7%, 95% CI: 50.5–66.9, P<0.01). The majority of the infections were attributed to Gram-negative bacteria, accounting for 101 out of 138 cases (73.2%, 95% CI: 65.6–80.7), with 69 out of 101 cases (68.3%, 95% CI: 63.8–72.8) involving ESBL-producing strains while Gram-positive bacteria were responsible for 26.8% (95% CI: 19.3–34.4) of the infections. Among the total sepsis cases, 72 out of 342 (21.1%, 95% CI: 16.7–25.4, P<0.01) experienced deaths attributed to sepsis. Within this group of deaths, 43 out of 72 (59.7%, 95% CI: 48.4–71.1, P<0.01) cases were linked to bacterial isolates in blood culture. In the subset of culture-positive deaths, 28 out of 72 (38.9%, 95% CI: 27.70–50.1, P<0.03) deaths were attributed to ESBL-producing bacteria, 9 out of 72 (12.5%, 95% CI: 5.3–19.8, P<0.01) deaths to non-ESBL-producing bacteria, and 6 out of 72 (8.3%, 95% CI: 1.9–14.8, P<0.01) deaths to Gram-positive bacteria (**Fig 1)**.

**Fig 2:**
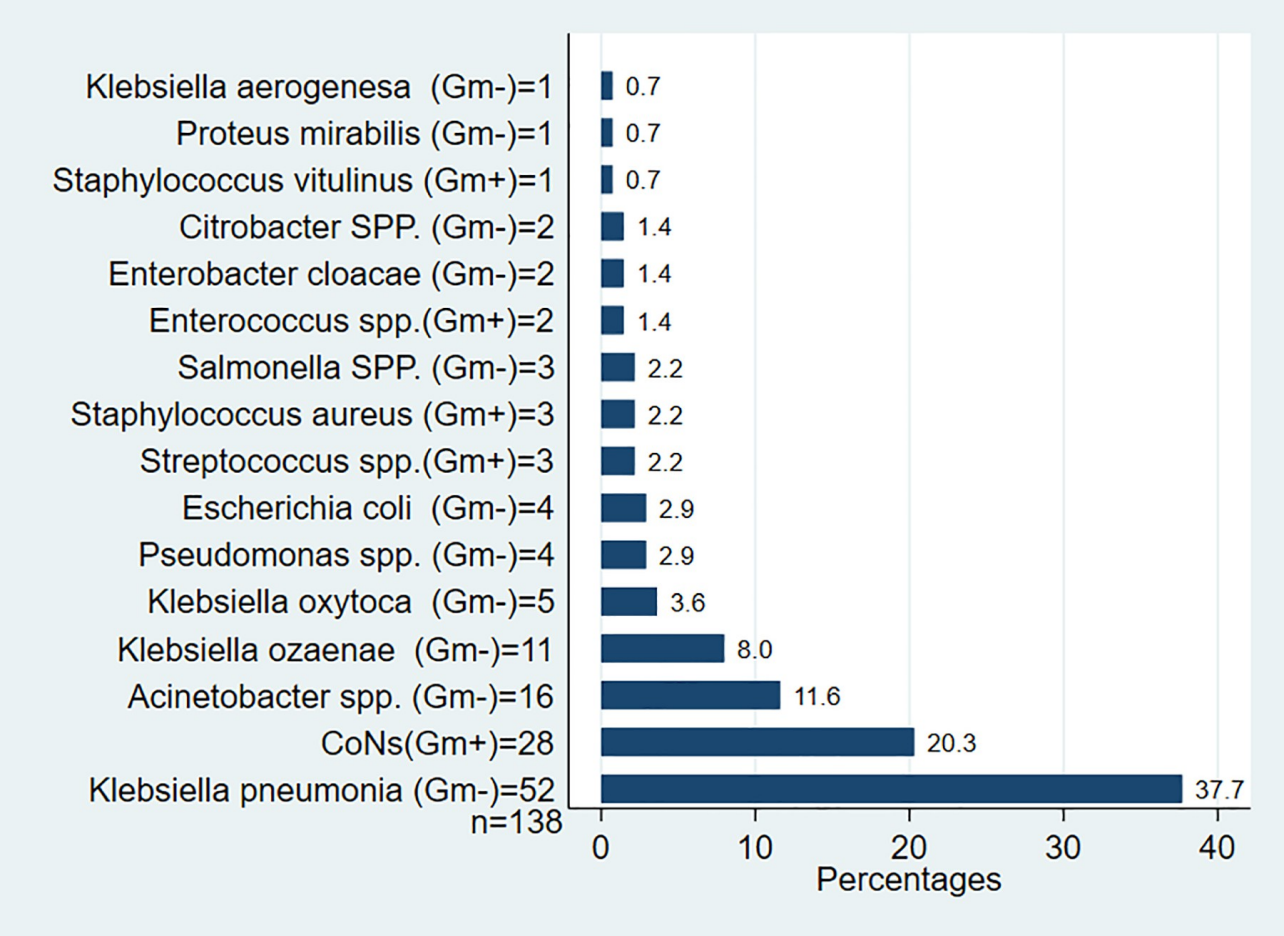
Distribution of bacteria isolated from neonatal blood sample at JMC, Ethiopia.

Gram-negative bacteria were identified in 101 out of 138 cases of sepsis, accounting for 73.2% (95% CI: 65.6–80.7), while Gram-positive bacteria were detected in 37 out of 138 cases, representing 26.8% (95% CI: 19.3–34.4). Among the Gram-negative bacterial species, the most commonly isolated ones were *K*. *pneumoniae*, Acinetobacter species, *Klebsiella ozaenae*, and *Klebsiella oxytoca*, found in 52 (37.7%, 95% CI: 29.6–45.8), 16 (11.6%, 95% CI: 6.0–17.1), 11 (8.0%, 95% CI: 4.0–12.0), and 5 (3.6%, 95% CI: 0.5–6.7) cases of neonatal sepsis, respectively. On the other hand, the prevalent Gram-positive bacteria included *CoNs* in 28 cases (20.3%, 95% CI: 13.6–27.0), Streptococcus species in 3 cases (2.2%), and *S*. *aureus* in 3 cases (2.2%, 95% CI: -0.3–3.1) as detailed by Fig 2.

### Antimicrobial resistance profiles of bacteria isolated from neonatal sepsis

The study conducted an analysis of resistance profiles in both Gram-negative and Gram-positive bacterial isolates, with the findings presented in Table 2. Among the Gram-negative isolates, *K*. *pneumoniae* (N = 52) exhibited resistance to a range of antibiotics, including SXT (100%, 52/52), CRO (100%, 52/52), CTX (98.1%, 51/52), CAZ (90.4%, 47/52), GEN (84.6%, 44/52), and MEM (1.9%, 1/52). Acinetobacter species (n = 16) also showed resistance patterns, notably to AMP (100%, 16/16), CTX (100%, 16/16), SXT (75%, 12/16), CAZ (68.8%, 11/16), CHL (68.8%, 11/16), CRO (68.8%, 11/16), and MEM (25%, 4/16). Similarly, *Klebsiella ozaenae* (n = 11) demonstrated resistance to several antibiotics, including AMP (100%, 11/11), SXT (100%, 11/11), CAZ (100%, 11/11), CTX (100%, 11/11), CRO (100%, 11/11), CIP (90.9%, 10/11), GEN (63.6%, 7/11), with no resistance observed to MEM. In the evaluation of Gram-positive bacterial strains, CoNs (n = 28) displayed resistance to various antibiotics, such as AMP (100%, 28/28), PEN (100%, 28/28), CTX (86.0%, 24/28), GEN (57.2%, 16/28), OXA (32.2%, 9/28), FOX (25.0%, 7/28), and VAN (10.7%, 3/28).

**Table 2:** Antibiotic resistance profiles of bacterial isolates (N = 138) in neonatal sepsis at JMC, Ethiopia.

Type of Isolates	Name and number of tested Isolates	Antibiotic classes and resistance: n (%)														
		PEN			BLI		SUL	PHE	QNL	AMN		CEP				CAR
		AMP	OXA	PEN G	TZP	AMC	SXT	CHL	CIP	GEN	VAN	FOX	CTX	CAZ	CRO	MEM
**Gram-negative**	*Klebsiella pneumoniae (N = 52)*	NRC	_	_	13(25.0)	30(57.7)	52(100)	29(55.8)	36(69.2)	44(84.6)	_	_	51(98.1)	47(90.4)	52(100)	1(1.9)
	*Acinetobacter spp*.*(N = 16)*	16(100)	_	_	8(50.0)	10(62.5)	12(75.0)	11(68.8)	9(56.3)	9(56.3)	_	_	16(100)	11(68.8)	11(68.8)	4(25.0)
	*Klebsiella ozaenae (N = 11)*	11(100)	_	_	5(45.5)	6(54.5)	11(100)	3(27.3)	10(90.9)	7(63.6)	_	_	11(100)	11(100)	11(100)	0
	*Klebsiella oxytoca (n = 5)*	5(100)	_	_	2(40.0)	3(60.0)	5(100)	3(60.0)	4(80.0)	3(60.0)	_	_	5(100)	5(100)	5(100)	0
	*Pseudomona spp(N = 4)*	4(100)	_	_	3(75.0)	3(75.0)	4(100)	3(75.0)	2(50.0)	3(75.0)	_	_	4(100)	3(75.0)	4(100)	0
	*Escherichia coli (N = 4)*	4(100)	_	_	3(75.0)	2(50.0)	2(50.0)	3(75.0)	2(50.0)	1(25.0)	_	_	4(100)	3(75.0)	4(100)	1(25.0)
	Others*(N = 9)	9(100)	_	_	3(33.3)	7(77.8)	8(88.9)	4(44.4)	8(88.9)	6(66.7)	_	_	9(100)	9(100)	9(100)	0
	**Total, n (%)**	**49(100)**	**_**	**_**	**37(36.6)**	**61(60.4)**	**94(93.1)**	**56(55.5)**	**72(71.3)**	**73(72.3)**	**_**	**_**	**100(99.0)**	**89(88.1)**	**96(95.0)**	**6(5.9)**
**Gram-positive**	*Staphylococcus*, *coagulase negative (N = 28)*	28(100)	16(57.1)	28(100)	_	_	_	_	_	16(57.2)	3(10.7)	7(25.0)	24(86.0)	_	_	_
	*Staphylococcus aureus (N = 3)*	3	0	3	_	_	_	_	_	2	0	0	2	_	_	_
	*Enterococcus spp(N = 2)*	2	0	1	_	_	_	_	_	2	0	0	2	_	_	_
	*Streptococcus spp(N = 3)*	3	0	2	_	_	_	_	_	1	2	0	0	_	_	_
	*Staphylococcus vitulinus(N = 1)*	1	0	1	_	_	_	_	_	0	1	0	1	_	_	_
**Overall**	**Total (n (%))**	**86(100)**	**16(57.1)**	**35(94.6)**	**37(36.6)**	**61(60.4)**	**94(93.1)**	**56(55.5)**	**72(71.3)**	**94(68.1)**	**6(16.2)**	**7(18.9)**	**129(93.5)**	**89(88.1)**	**96(95.0)**	**6(5.9)**

Note: N = Number of isolates undergone antibiotic susceptibility test; n = number of isolated became resistant to particular antibiotic; NRC = Not recommended, (_) = antibiotic susceptibility test not done; Others

* referred for Salmonella SP ((N = 3), Citrobacter (N = 2), Enterobacter cloacae spp.(N = 2),*Klebsiella aerogenesa* (N = 1) and Proteus mirabilis(N = 1); the abbreviated antimicrobial names and classes were detailed in the methodology section. Susceptible (S): organisms are inhibited by standard dosages of the antimicrobial agent, Intermediate (I): exist in a transitional state, and, and Resistant (R) demonstrate the ability to withstand the effects of the antimicrobial agent to which they are previously susceptible. Ampicillin (AMP), Benzyl Penicillin (PEN), Oxacillin (OXA), Sulfamethoxazole-trimethoprim (SXT), Amoxicillin-Clavulanate (AMC), Tazobactam (TZP), Chloramphenicol (CHL), Ciprofloxacin (CIP), Gentamicin (GEN), Vancomycin (VAN), Cefotaxime (CTX), Ceftazidime (CAZ), Ceftriaxone (CRO), Cefoxitin (FOX), and Meropenem (MEM).

### Prevalence of ESBL and non-ESBL-producing bacterial isolates in neonatal sepsis

**Fig 3:**
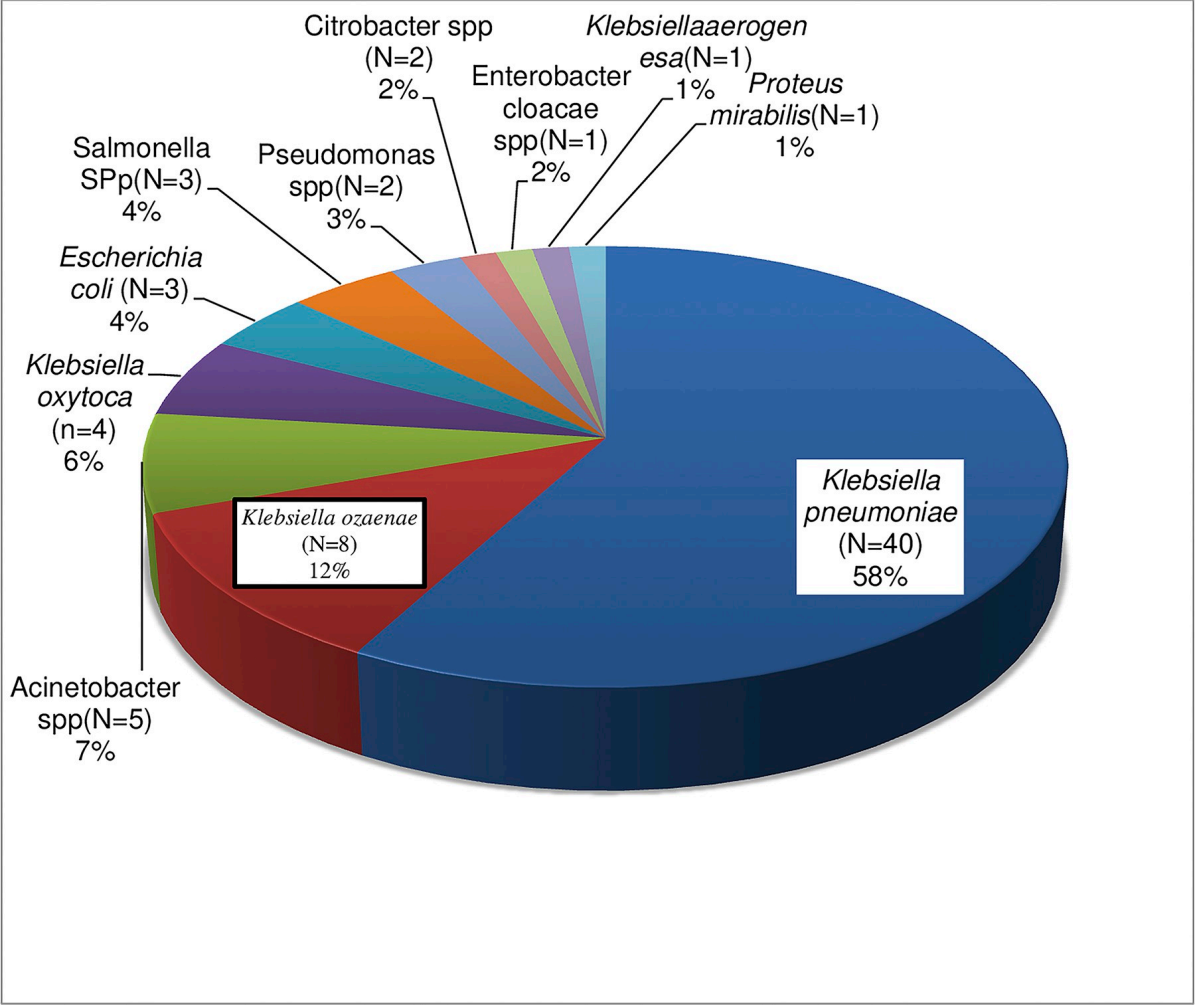
The prevalence of ESBL producing Gram-negative isolates among neonates with sepsis at JMC, Ethiopia.

The characterization of Gram-negative bacterial isolates revealed that 69/101 (68.3%, 95% CI: 63.8–72.8) were identified as ESBL producers, with the remaining 31.7% (95% CI: 22.6–40.7) isolates not exhibiting ESBL production. Within the ESBL-producing isolates, *K*. *pneumoniae* represented 58.0% (40/69), followed by *K*. *ozaenae* at 12.0% (88/69), Acinetobacter spp. at 7.0% (5/69), and *K*. *oxytoca* at 6.0% (4/69). The remaining 17% (12/69) comprised *E*. *coli*, Salmonella spp., Pseudomonas spp., Citrobacter spp., Enterobacter cloacae spp., *Klebsiella erogenesa*, and *Proteus mirabilis* (Fig 3).

### Antimicrobial resistance profiles of ESBL and Non-ESBL producing isolates

**Fig 4:**
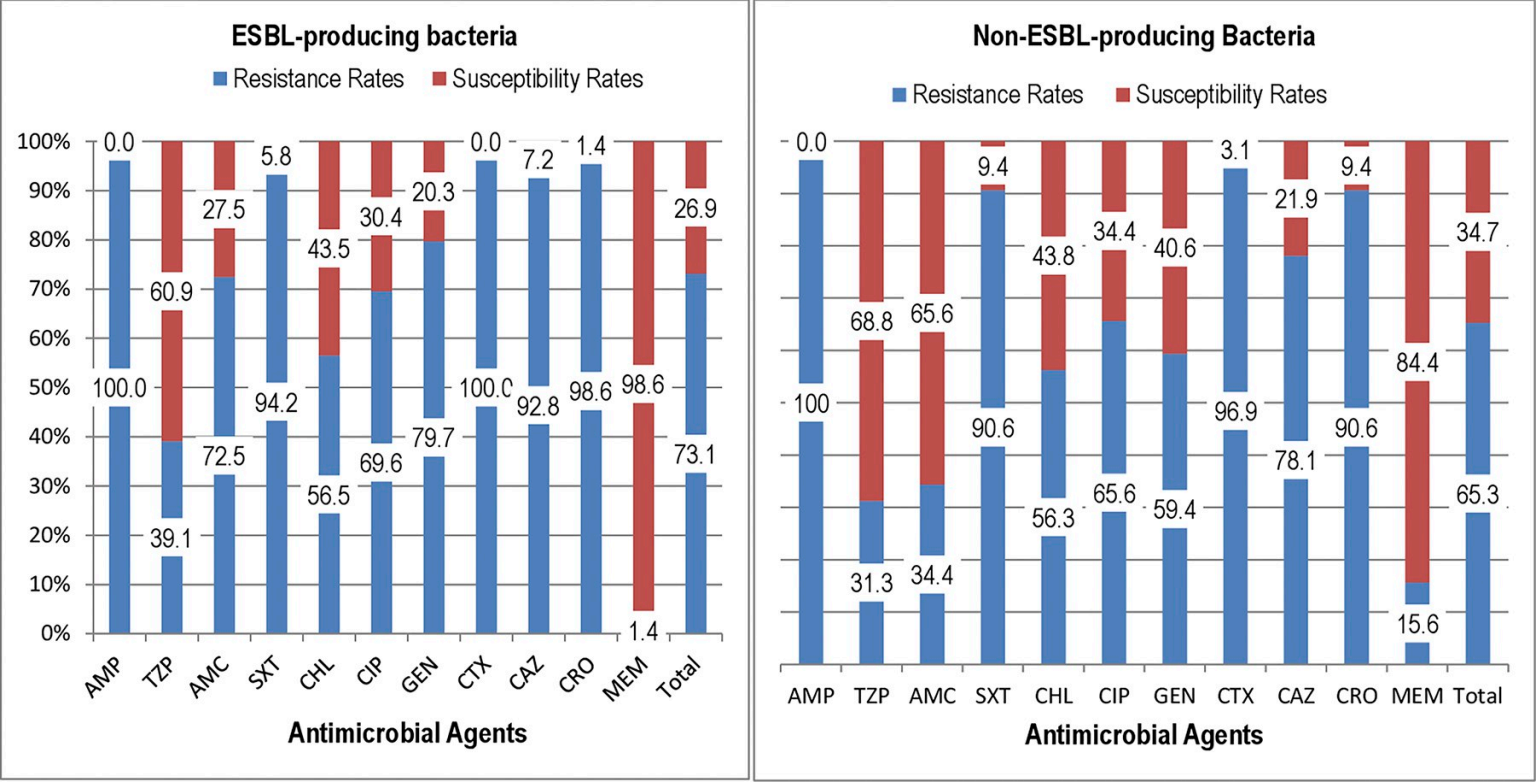
Comparative Antimicrobial Resistance Profiles of ESBL (left) and Non-ESBL (right) Producing isolates in neonatal sepsis at JMC, Ethiopia.

The study had identified the overall independent resistance rates of ESBL-producing bacteria 555/759 (73.1%, 95% CI: 69.9–76.1) and non-ESBL-producing bacteria 230/352 (65.3%, 95% CI: 60.1–70.2) to various antibiotics. ESBL-producing isolates did exhibit higher rates of resistance compared to non-ESBL-producing isolates, except for AMP, CHL, and MEM. The ESBL-producing isolates showed a similar resistance rate to CHL at 56.3% vs. 56.5% and lower resistance to MEM at 1.4% vs 15.6% compared to non-ESBL-producing isolates. The resistance profiles revealed that most of the ESBL-producers were resistant to first line antibiotic regimen [AMP (100%), CTX (100%) or GEN (79.7%)] used to treat infection (Fig 4).

### Multidrug resistance profiles of bacterial isolates in neonatal sepsis

The study revealed that 122 out of 138 bacterial isolates (88.4%, 95% CI: 81.8%-93.0%) were resistant to at least one antibiotic in three or more drug classes, making them MDR. Specifically, 19 (13.8%), 24 (17.4%), 26 (18.8%), 22 (15.9%), and 31 (22.5%) bacterial isolates were resistant to three, four, five, six, and seven classes of antibiotics, respectively. Among the various bacterial isolates, all *K*. *pneumoniae* and exhibited MDR, while Acinetobacter spp., and *CoNs* showed rates of 93.8%, and 67.9% MDR rate, respectively. The details of other bacterial MDR profiles were presented in Table 3.

**Table 3:** Prevalence of MDR in bacterial isolates in neonatal sepsis at JMC, Ethiopia.

Bacterial Isolate	Degree of resistance								Total MDR≥R_3_
	R_0_	R_1_	R_2_	R_3_	R_4_	R_5_	R_6_	R_7_	
*K*.*pneumoniae (N = 52)*	0	0	0	0	7	15	16	14	52(100)
*Acinetobacter spp*.*(N = 16)*	0	0	1	2	4	1	1	7	15(93.8)
*K*. *ozaenae (N = 11)*	0	0	0	1	2	4	3	1	11(100)
*K*.*oxytoca (n = 5)*	0	0	0	1	1	0	0	3	5(100)
*Pseudomona spp(N = 4)*	0	0	1	0	0	1	1	1	3(75)
*E*. *coli (N = 4)*	0	0	0	0	1	1	0	2	4(100)
Others*(N = 9)	0	0	0	0	1	4	1	3	7(100)
*CoNs (N = 28)*	0	5	4	11	8	0	0	0	19(67.9)
*Others* **	0	2	3	4	0	0	0	0	4(44.4)
***Total (n = 138)***	**0**	**7(5.1)**	**9(6.5)**	**19(13.8)**	**24(17.4)**	**26(18.8)**	**22(15.9)**	**31(22.5)**	**122(88.4)**

Note: R_0_-No antibiotic resistance, R_1_-Resistance to one classes of antimicrobial agent, R_2-_ Resistance to two different classes of antimicrobial agent, R^3^- Resistance to three different classes of antimicrobial agent, R_4_ -Resistance to four different classes of antimicrobial agent, R_5_- Resistance to five different classes of antimicrobial agent, R_6_- Resistance to six different classes of antimicrobial agent, R_7_-Resistance to seven different classes of antimicrobial agent.; Others

* referred for Salmonella SP ((N = 3), Citrobacter (N = 2), Enterobacter cloacae spp.(N = 2),*Klebsiella aerogenesa* (N = 1) and *Proteus mirabilis*(N = 1); Others

** referred for *Staphylococcus aureus* (N = 3), Enterococcus spp(N = 2), Streptococcus spp(N = 3) and *Staphylococcus vitulinus*(N = 1)

The majority of Gram-negative bacteria 99/101 (98.0%) exhibited MDR characteristics, with 68/69 (98.6%, 95% CI: 92.1–100.0) of ESBL-producing isolates and 31/32 (96.9%, 95% CI: 83.8–99.9%) of non-ESBL-producing isolates demonstrating MDR. ESBL-producing Gram-negative isolates contributed to the overall MDR at a rate of 68/138 (49.3%, 95% CI: 45.1%-53.5%), while non-ESBL-producing Gram-negative isolates contributed at a rate of 31/138 (22.5%, 95% CI: 18.9%-26.0%). Furthermore, 23/37 (62.2%, 95% CI: 45.1–77.1) of Gram-positive bacteria exhibited MDR, contributing 23/138 (16.6%, 95% CI: 13.4%-19.9%) to the overall MDR rate. The most frequent MDR pattern observed across all bacterial isolates involved resistance to seven different classes of antibiotics (PEN/BLI/SUL/PHE/QNL/AMN/CEP) and was detected in 28/122 (23.0%, 95% CI: 15.8–31.6) of the total isolates. The second most common pattern consisted of resistance to four classes of antibiotics (PEN/SUL/AMN/CEP) covering 12/122 (99.8%, 95% CI: 5.2–16.6) of the isolates. Among the Gram-negative isolates, those producing ESBL exhibited a wide range of MDR patterns, while non-ESBL-producing isolates displayed a more consistent distribution of the MDR pattern. In contrast, Gram-positive bacteria typically showed MDR patterns that involved resistance to three to four classes of antibiotics. The three-class resistance patterns observed in Gram-positive isolates included resistance to PEN/SUL/CEP and PEN/AMN/SUL, while the four-class resistance pattern involved resistance to PEN/SUL/AMN/CEP (Table 4).

**Table 4:** MDR patterns and contribution of bacterial isolates in neonatal sepsis (N = 138) at JMC, Ethiopia.

Antibiotic Classes and Patterns of resistance	Gram-negative (n = 101)		Gram-positive (n = 37)	Total (n = 138)(%)
	ESBL-producers (n = 69)	Non-ESBL-producers (n = 32)		
PEN/BLI/CEP	1	0	0	1(0.8)
PEN/SUL/CEP	2	1	5	8(6.6)
PEN/SUL/AMN	0	0	10	10(8.2)
PEN/SUL/AMN/CEP	2	2	8	12(9.8)
PEN/SUL/QNL/CEP	2	2	0	4(3.3)
PEN/BLI/SUL/CEP	2	1	0	3(2.5)
PEN/SUL/PHE/CEP	0	4	0	4(3.3)
PEN/BLI/PHE/CEP	0	1	0	1(0.8)
PEN/BLI/SUL/AMN/CEP	3	2	0	5(4.1)
PEN/SUL/PHE/AMN/CEP	3	1	0	4(3.3)
PEN/SUL/QNL/AMN/CEP	6	4	0	10(8.2)
PEN/BLI/SUL/QNL/CEP	4	0	0	4(3.3)
PEN/BLI/SUL/PHE/CEP	1	0	0	1(0.8)
PEN/BLI/QNL/AMN/CEP	1	0	0	1(0.8)
PEN/SUL/PHE/QNL/CEP	0	1	0	1(0.8)
PEN/PHE/QNL/AMN/CEP	0	1	0	1(0.8)
PEN/BLI/PHE/QNL/AMN/CEP	1	0	0	1(0.8)
PEN/BLI/SUL/PHE/QNL/CEP	3	0	0	3(2.5)
PEN/BLI/SUL/PHE/AMN/CEP	6	1	0	7(5.7)
PEN/BLI/SUL/QNL/AMN/CEP	8	1	0	9(7.4)
PEN/SUL/PHE/AMN/CEP/CAR	0	1	0	1(0.8)
PEN/BLI/SUL/PHE/QNL/CEP/CAR	1	1	0	2(1.6)
PEN/BLI/SUL/PHE/AMN/CEP/CAR	0	1	0	1(0.8)
PEN/BLI/SUL/PHE/QNL/AMN/CEP	22	6	0	28(23.0)
**Total**	**68(49.3%)**	**31(25.4)**	**23(18.9)**	**122(88.4)**

Note: PEN: Penicillin, BLI: Beta-lactamase inhibitors, SUL:Sulfonamide, PHE: Phenols, QNL: Fluoroquinolones, AMN: Aminoglycosides, CEP: Cephalosporins, and CAR: Carbapenems.

## Discussion

A cohort study conducted in a tertiary healthcare setting in Ethiopia examined the phenotypic epidemiology, antimicrobial resistance profiles, and clinical significance of bacterial isolates in 342 neonates. The study revealed a significant prevalence of blood culture-confirmed neonatal sepsis and associated mortality among neonates suspected of clinical sepsis. Gram-negative bacteria were identified as the primary causative agents of neonatal sepsis, contributing to high levels of antimicrobial resistance among the isolates.

Specifically, this study publicized a concerning 40.6% prevalence rate of confirmed neonatal sepsis and a 21.1% associated mortality rate, disproportionately affecting male neonates and those under three days old, similar to findings from Nepal and Jimma where it mostly occurred male neonates [30, 31]. The overall 40.6% prevalence rate, however, surpasses the prevalence rates reported, India (14.6%) [32], Indonesia (15.3%) [33], Nepal (16.9%) [30], Tanzania (21.2%) [34], the Central African Republic (26.6%) [35], and various areas in Ethiopia such as Addis Ababa (21%) [23], Hawasa (36.5%) [36], Wallo (27.2%) [37], Assela (29.0%) [38], Tigray (36.6%) [39], and a multicenter study in Ethiopia (37.0%) [40] and previous study setting (23.7%) [31]. However, the current finding agrees with the elevated rates observed in neighboring countries like Egypt (40.7%) [12] and a domestic area in Ethiopia; Bahirdar (41.3%) [41]. Conversely, the current study prevalence remained lower than the considerably higher sepsis rates reported in Tanzania (72%) [22] and India (57.5%) [42], as well as certain studies conducted in Ethiopia like Gondar (46.6%) [43], Adama (46.4%) [44] and the previous study result of the current study setting, Jimma (58.0%) [45]. Likewise, the associated mortality rate exceeded the 11.3% global mortality rate [46], 10.7% in Tigray [39], and the previous study in the same setting (12.2%) [45] and (16.5%) [31], but was lower than the results reported from Tanzania (37.2%) [34] and Addis Ababa (24.4%) [47]. Notably, the comparison of results highlighted substantial temporal and spatial heterogeneity in morbidity and mortality attributed to neonatal sepsis where literatures claim the reason for heterogeneity could be iatrogenic risks, neonatal diversity, methodological differences, detection capacity, and existing infection control measures [48].

The analysis of bacterial isolates conducted in the current study demonstrated that Gram-negative bacteria accounted for 73.2% of the cases, whereas Gram-positive bacteria constituted only 26.8% of the cases. These findings are in accordance with previous studies indicating that Gram-negative bacteria are the primary etiological agents of sepsis in various regions, such as Tanzania (over 50%) [20], Bahardar (60.0%) [41], Hawasa (62.7%) [36], and Adama (69.9%) [44]. In contrast, the results of this study contradict earlier findings that identified Gram-positive bacteria as the predominant cause of neonatal sepsis in countries like Germany (74%) [7], India (50%) [32],Tanzania (81%) [22],Gondar (67.5%) [43] and Jimma [45]. Particularly, the most frequently isolated bacterial species in this study included *K*. *pneumoniae* (37.7%), *CoNs* (20.3%), Streptococcus species (8.1%), and Acinetobacter species (8.0%). Furthermore, concerning specific pathogens, this study exhibited similarities with research conducted in Zambia (74%) [20], Adama (48.98%) [44], Tigray (35%) [39], Bahardar (28.2%) [41], and Hawasa (47.4%) [36], which identified *K*.*pneumoniae* as the most prevalent pathogen. It also concurs with reports emphasizing the predominance of *CoNs* among Gram-positive bacteria, where it reported 52.9% in Egypt [12], 14.97% in Adama [44], 25% in Arsi [38], 9.4% in Tigray [39], and 25.7% in Jimma [45]. However, the findings of the current study contrast with the reported prevalence rates of *Staphylococcus aureus* (24.7% in Bahardar [41] and 40.8% in Gondar [43] and *E*. *coli* (35.8% in Wallo) [37]. The observed variations in the prevalence and distribution of neonatal sepsis-causing bacteria are classically attributed to differences in study populations, local epidemiology, study methodologies, diagnostic techniques, neonatal care practices, antibiotic utilization, and regional microbial diversity [48].

The antibiotic resistance profile of the isolates revealed significant variability, ranging from 5.9% for meropenem to 100% for ampicillin. The highest resistance rates were observed for ampicillin at 100%, ceftriaxone at 95%, penicillin at 94.6%, cefotaxime at 93.5%, and ceftazidime at 88.1%, consistent with studies conducted in regions such as Egypt [49] and parts of Ethiopia including Bahirdar [41] and Hawasa [36]. Gram-negative bacteria exhibited alarmingly high resistance to ampicillin at 100%, cefotaxime at 99.0%, ceftriaxone at 95.0%, sulfamethoxazole-trimethoprim at 93.1%, and ceftazidime at 88.1%. The resistance data revealed Klebsiella pneumoniae with the highest percentage of resistant isolates, showing resistance to sulfamethoxazole-trimethoprim at 100%, ceftriaxone at 100%, cefotaxime at 98.1%, and gentamicin at 84.6%, followed closely by Acinetobacter spp. with 100% resistance to ampicillin and cefotaxime, and 68.8% to gentamicin. *Coagulase-negative Staphylococci* also showed high resistance to ampicillin, penicillin G, and cefotaxime at 86%. Characteristically, the dominance of Gram-negative bacteria in the resistant profile resembles previous study results across several countries, including German [7], India [32], Tanzania [22], China [19], India [42], and Ethiopia in Addis Abeba [23]. The possible reason for the dominance of Gram-negative bacteria in resistant profiles might be linked to various mechanisms of drug resistance. Specifically, Gram-negative bacteria possess an outer membrane that acts as a physical barrier, making it difficult for antibiotics to penetrate the cell wall [50]. They also have efflux pumps that actively remove antibiotics from the cell, diminishing the effectiveness of these drugs, while some harbor plasmids carrying genes for resistance [51]. Additionally, these bacteria produce beta-lactamase enzymes, including ESBLs, which can degrade a broader range of beta-lactam antibiotics, thereby ensuring their dominance in antimicrobial resistance [52, 53].

In the context of ESBL-production, this study found that 68.3% of the bacterial isolates analyzed exhibited ESBL activity. Notably, *K*. *pneumoniae* was the most prevalent strain at 58.0%, followed by *Klebsiella ozaenae* at 12.0% (8 out of 69), Acinetobacter species at 7.0%, and *Klebsiella oxytoca* at 6.0%. The prevalence of ESBL-producing isolates in this study was higher than the rates observed in Tanzania [54] and 54.0% from a prior Ethiopia study [21]. However, the isolate distribution is consistent with reports in Tanzania [22], Nepal [30], China [19], India [42], and prior result in Ethiopia [23], which also highlighted the dominance of ESBL-producing Gram-negative bacteria in neonatal sepsis. Further, it was observed that ESBL-producing strains exhibited significantly higher resistance levels to commonly prescribed antibiotics (Piperacillin-tazobactam, Amoxicillin-clavulanate, Sulfamethoxazole-trimethoprim, Ciprofloxacin, Gentamicin, Cefotaxime, Ceftazidime, and Ceftriaxone) compared to non-ESBL producers resembling the report in Pakistan [11] and Tanzania [55]. The study found that ampicillin resistance was identical across all ESBL-producing and non-ESBL-producing strains, suggesting a common mechanism. However, non-ESBL-producing strains exhibited significantly higher resistance levels to chloramphenicol and meropenem antibiotics compared to ESBL-producing strains. This could be due to the presence of additional beta-lactamase enzymes beyond ESBLs, such as carbapenem-hydrolyzing Klebsiella pneumoniae carbapenemase (KPC), metallo-beta-lactamase New Delhi metallo-beta-lactamase (NDM), and variants of the oxacillinase beta-lactamase class [56]. The clinical importance of ESBL-producing bacteria is highlighted in the literature, particularly due to the increasing virulence of Gram-negative pathogens in vulnerable groups like neonates. This poses a significant transmission risk in healthcare settings, compounded by factors such as hospital-acquired infections, preterm births, cesarean deliveries, and low birth weights commonly observed in population under this study [21].

Finally, the study revealed a high prevalence of multidrug resistance (MDR) in isolated bacteria, with an occurrence rate of 88.4% among all isolates. This rate is slightly higher than that in China (78.3%) [19] and a previous study in Ethiopia (84%) [23]. Notably, Gram-negative strains showed a higher MDR rate at 98.0% compared to Gram-positive strains at 62.2%, consistent with earlier findings in Ethiopia [57]. Moreover, Gram-negative isolates producing ESBLs had a MDR rate of 98.6%, highlighting the challenge in treating ESBL-producing bacteria. ESBL-producing Gram-negative bacteria accounted for 49.3% of MDR cases, aligning with previous study results [58]. ESBL-producing isolates displayed more diverse patterns of multidrug resistance across a wider range of antibiotics compared to non-ESBL-producing Gram-negative and Gram-positive bacteria. The dominance of Gram-negative bacteria, especially with ESBL production, corresponds with findings from Tanzania [59]. The resilience of MDR Gram-negative bacteria can be attributed to their complex outer membrane structure, efflux pumps, porin channels, and advanced antibiotic targets, further enhanced by ESBL enzyme production. The variations in study results are expected due to the interplay of factors favoring resistance in Gram-negative bacteria, which interact with a variety of other variables operating differently for Gram-positive bacteria. These variables likely influence the prevalence of bacterial antimicrobial resistance across geographical regions, healthcare settings, and study populations, leading to distinct results [17].

In general, bacteria epidemiology and resistance profiles are influenced by various factors, causing temporal and spatial variability. To tackle this issue, our recent study utilized larger sample sizes, an observational approach, and a certified laboratory to minimize these variables’ impact. We employed randomized participant selection and a prospective design, conducting blood culture tests at a single study center for validity. By focusing on a single research center, we could control variables such as location, staff, resources, and procedures. However, it’s essential to carefully consider the limitations of this approach when inferring the findings to the broader population beyond the study setting.

## Conclusion and recommendation

The study has identified a high prevalence of confirmed sepsis cases and associated mortalities among neonates presenting with clinically suspected sepsis. Pathogens such as *Klebsiella pneumoniae*, *Coagulase-negative Staphylococci*, Acinetobacter species, and *Klebsiella ozaen*ae primarily influence the epidemiology of neonatal sepsis by producing Extended-Spectrum Beta-Lactamases (ESBLs) in Klebsiella and Acinetobacter species, leading to antimicrobial resistance and multidrug resistance (MDR). These multidrug-resistant strains pose a significant challenge to current treatment approaches, necessitating the prompt development of innovative empiric antibiotic regimens. The findings of the study emphasize the critical importance for neonatal intensive care units to prioritize infection prevention strategies, reliable diagnostic stewardship, tailored antimicrobial stewardship programs, and well-informed decisions based on surveillance data. Further research is vital to elucidate the factors driving widespread antibiotic resistance, particularly in identifying resistance genes in ESBL-producing isolates, to guide the development of strategies to combat this escalating public health threat.

## Supporting information

S1 Data (CSV)

## Lists of abbreviations

CI: Confidence Interval
CoNS: Coagulase-negative Staphylococcus
ESBL: Extended-Spectrum Beta-Lactamase
GBS: Group B Streptococcus
JMC: Jimma Medical Center
MDR: Multi drug resistance
NICU: Neonatal Intensive Care Unit
